# Intravenous ferric derisomaltose versus saccharated ferric oxide for iron deficiency anemia associated with menorrhagia: a randomized, open-label, active-controlled, noninferiority study

**DOI:** 10.1007/s12185-022-03401-0

**Published:** 2022-07-06

**Authors:** Hiroshi Kawabata, Takeshi Tamura, Soichiro Tamai, Akiko Fujibayashi, Motoi Sugimura, Study Group, Jun Hayakawa, Hisato Oku, Yoshiaki Ota, Sonoe Nishiguchi, Kiyohiko Yamada, Masayasu Nomura, Toshiro Mizutani, Yoshihiro Tamura, Kyoka Amemiya, Mamoru Urabe, Hirofumi Henmi, Kozo Aisaka, Atsuya Fujito, Masataka Oku, Chisei Tei, Akinori Kawata, Masaya Hirose, Masuo Yoshioka, Chizue Nishizawa, Ikuyo Horiguchi, Kozo Hirai, Akiko Tanabe, Shohei Yoshida, Yoshihiro Umezawa, Yuji Kashiwazaki, Hideki Kamegai, Toshio Saito, Kazutoshi Naritaka, Shigehito Yamauchi, Kenji Akazawa, Koji Kobiki, Hiroshi Tsujioka, Yukari Sumi, Reiko Matsumoto, Mari Kiuchi, Yukari Utsugisawa, Masanori Maruyama, Hiroyuki Furumoto, Kazuhiro Minegishi, Masao Takane, Asuka Yoshii, Tsuneo Yokokura, Hideki Hanashi, Sumie Yukawa

**Affiliations:** 1 Department of Hematology, National Hospital Organization Kyoto Medical Center, Kyoto, Japan; 2 Clinical Development Department, Nippon Shinyaku Co., Ltd, Kyoto, Japan; 3 Data Science Department, Nippon Shinyaku Co., Ltd, Kyoto, Japan; 4 Department of Obstetrics, Gynecology and Family Medicine, Hamamatsu University School of Medicine, Hamamatsu, Shizuoka Japan

**Keywords:** Ferric derisomaltose, Iron deficiency anemia, Intravenous iron preparation, Hypophosphatemia

## Abstract

A multicenter, randomized, open-label, phase III study was conducted to compare the efficacy and safety of intravenous ferric derisomaltose (FDI) versus saccharated ferric oxide (SFO) in Japanese patients with iron deficiency anemia associated with menorrhagia. FDI can be administered as a single dose up to 1000 mg, whereas SFO has a maximum single dose of 120 mg. The primary endpoint, which was the maximum change in hemoglobin concentration from baseline, was noninferior for the FDI group compared with the SFO group. The incidence of treatment-emergent adverse events was lower in the FDI group (66.2%) than in the SFO group (90.8%). Notably, the incidence of serum phosphorus level < 2.0 mg/dL was significantly lower in the FDI group (8.4%) than in the SFO group (83.2%), and severe hypophosphatemia (≤ 1.0 mg/dL) occurred in 6.7% of SFO‑treated patients compared with none in the FDI group. The percentage of patients who achieved the cumulative total iron dose during the 8-week treatment period was higher in the FDI group (92.8%) than in the SFO group (43.2%). The study met its primary endpoint, and also demonstrated the tolerability of a high dose of FDI per infusion, with a lower incidence of hypophosphatemia.

**Supplementary Information:**

The online version contains supplementary material available at 10.1007/s12185-022-03401-0.

## Introduction

Iron deficiency anemia (IDA) is the most common anemia, impacting more than 1 billion individuals [1]. The major causes of IDA include menorrhagia and uterine myoma (or fibroids) in premenopausal women and gastrointestinal bleeding in men and postmenopausal women; other causes include chronic kidney disease, malignancies, congestive heart failure, and inflammatory bowel disease [2, 3]. Among Japanese women, the major causes of IDA are vaginal bleeding [35.9%, including menorrhagia (17.9%) and uterine fibroids (14.6%)] and gastrointestinal bleeding (11.7%) [4]. Guideline-based therapies for IDA include treatment of its underlying causes and the restoration of adequate iron stores [5]. Oral iron supplementation is the first-line therapy for iron restoration; however, 10%–20% of patients experience gastrointestinal side effects, such as nausea, constipation, abdominal pain, diarrhea, and vomiting. Moreover, some patients are refractory to oral iron preparations due to severe menorrhagia or poor drug adherence. In such cases, intravenous (IV) iron supplementation should be considered.

When this study was initiated, the only available IV iron preparation in Japan was saccharated ferric oxide (SFO). The maximum daily dose of SFO is 120 mg. Guidelines recommend the injection of 40–120 mg of iron on consecutive days until the cumulative dose is achieved [5]. Hence, potentially many infusions of SFO are required to correct IDA, resulting in frequent treatment visits leading to patient inconvenience. In addition to these limitations on dosing, SFO increases fibroblast growth factor-23 (FGF23), which can lead to hypophosphatemia, biochemical changes in bone turnover [6], and an increased risk of osteomalacia [7–9].

Ferric derisomaltose (FDI), also known as iron isomaltoside 1000 (Pharmacosmos A/S [Denmark]), is an IV iron preparation approved for iron deficiency in Europe since 2009 and is currently marketed worldwide [10]. FDI contains oligosaccharides with a molecular weight of approximately 1000 Da and is a linear and unbranched structure consisting predominantly of 3–5 glucose units with a low immunological potential. Owing to the slow release of labile iron from the carbohydrate backbone [11–13], FDI is considered to have a favorable toxicity profile in IDA [14–16]. Its higher stability, suitable molecular weight, and low risk of free iron-related toxicities enable a rapid IV infusion of high-dose iron. As either a single bolus injection or an infusion, FDI was found to be well tolerated by Japanese patients with IDA in a phase I study (ClinicalTrials.gov identifier: NCT03013439).

Expanding upon earlier research, this study aimed to examine the efficacy and safety of FDI versus SFO in patients with IDA associated with menorrhagia.

## Materials and methods

### Study design

We conducted a multicenter, randomized, open-label, active-controlled phase III study to examine the noninferiority of the efficacy of FDI to SFO in patients with IDA associated with menorrhagia at 41 study sites in Japan from February 2019 to December 2019. The protocol and the informed consent forms (ICFs) were approved by the Institutional Review Board (IRB) at each participating site according to Good Clinical Practice (GCP) guidelines. This study was registered in the Japic Clinical Trials Information as JapicCTI-194573 and conducted in accordance with the ethical principles of the Declaration of Helsinki (October 2013) and GCP as well as the study protocol. Before any study procedure, the ICF was signed by both the patient and the person who conducted the informed consent discussion.

### Patients

This study was designed for a 2:1 randomization to either FDI or SFO. Approximately 360 patients were stratified by hemoglobin (Hb) concentration at the time of informed consent [screening visit (Vs)] and body weight at week 0, both important variables for total iron dose determination. Blinding for this study was not operationally feasible owing to substantial differences in the number of doses between FDI and SFO.

Japanese females between 18 and 49 years of age were considered to be eligible at the primary registration if they were diagnosed with IDA associated with menorrhagia and had been intolerant to oral iron preparations for the previous 2 years, failed to respond to oral iron preparations for ≥ 1 month, or were considered to require immediate iron supplementation by the site principal investigator. Final eligibility criteria, determined at the screening visit, were Hb < 11.0 g/dL, serum ferritin < 12 ng/mL, and a total iron-binding capacity ≥ 360 μg/dL. The main exclusion criteria were anemia due to causes other than iron deficiency, iron overload or defective iron utilization, acute or chronic infection, risks of increasing severity of hypersensitivity, being pregnant or nursing, or a history of allergy to any components of either the FDI or SFO preparation.

### Interventions

**Fig. 1:**
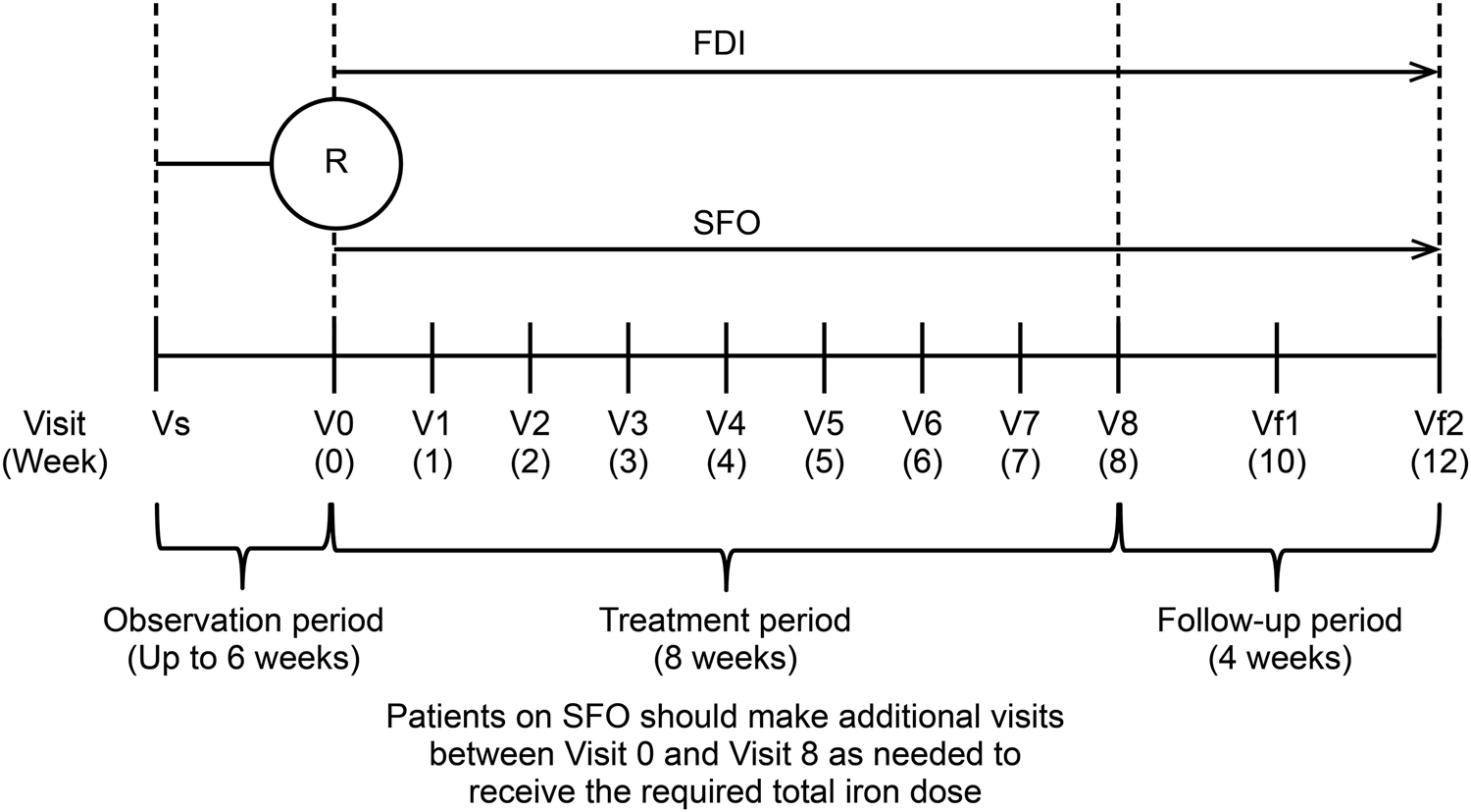
Study design in Japanese female patients with iron deficiency anemia associated with menorrhagia. *FDI* ferric derisomaltose, *R* randomization, *SFO* saccharated ferric oxide, *V* visit, *Vs* screening visit, *Vf1* follow-up visit 1, *Vf2* follow-up visit 2.

The study consisted of 3 study periods: an observation period of up to 6 weeks (including 1 menstrual cycle) before treatment initiation, an 8-week treatment period with mandatory visits occurring once a week, and a 4-week follow-up period (Fig. 1). For both the FDI and SFO treatment groups, the total iron dose was determined using the Hb concentration and body weight according to a simplified table (Table 1). For patients weighing < 40 kg, the dose was calculated using the Uchida formula [(2.2 × (16-pre-dose patient Hb concentration [g/dL]) + 10) × body weight (kg)] [17].

**Table 1:** Simplified table

Pre-dose Hb concentration (g/dL)	Weight < 40 kg	Weight 40 to < 50 kg	Weight 50 to < 70 kg	Weight ≥ 70 kg
≥ 10	Uchida formula^a^	750 mg	1000 mg	1500 mg
< 10	Uchida formula^a^	1000 mg	1500 mg	2000 mg

(The total iron dose was rounded to integers)

*Hb* hemoglobin

^a^Uchida formula: [2.2 × (16-pre-dose patient Hb concentration [g/dL]) + 10] × body weight (kg)

#### Ferric derisomaltose dosing

For the FDI group, the first dose was administered at week 0 (day 1) by IV drip infusion for all patients. For patients weighing ≥ 50 kg, the first FDI dose was 1000 mg. For patients weighing < 50 kg, the dose was calculated at 20 mg/kg of body weight. If the total iron dose was not fully administered as the first dose, the difference was administered at week 1 (between days 8 and 10) as the second dose. If the second dose was ≤ 500 mg, it was administered as a bolus; if > 500 mg, it was administered by drip infusion. For drip infusion, FDI was diluted with physiological saline ≤ 500 mL and administered over 15 min. As a bolus, FDI was either undiluted or diluted with physiological saline of ≤ 20 mL and administered over 2 min.

#### Saccharated ferric oxide dosing

For the SFO group, the first dose was administered at week 0 (day 1). The maximum daily dose was 120 mg by IV injection over 2 min. SFO was either undiluted or diluted 5–10 times with a 10%-20% glucose solution. In between the protocol-specified visits, additional visits were required for the total iron dose to be administered before week 8.

#### Treatment interruption criteria for serum phosphorus and ferritin levels

Treatment was interrupted if serum phosphorus decreased to < 2.0 mg/dL or if serum ferritin increased to > 500 ng/mL. Dosing was resumed if serum phosphorus increased to > 2.5 mg/dL and serum ferritin decreased to < 250 ng/mL.

### Endpoints

The primary efficacy endpoint was the maximum change in Hb from baseline. Secondary endpoints were changes in iron-related biochemical parameters [i.e., Hb concentration, transferrin saturation (TSAT), and serum ferritin level] and cumulative iron dose achievement. The mean corpuscular volume (MCV) and reticulocyte ratio were additional efficacy endpoints. Change in serum hepcidin concentration was an exploratory endpoint. The safety endpoints were adverse events (including symptoms of hypersensitivity), clinical laboratory tests (hematology, serum chemistry, and urine tests), physical examination, vital signs (blood pressure and pulse rate), and standard 12-lead electrocardiography (ECG).

### Sample size determination

The sample size was calculated by testing for noninferiority of the primary endpoint between the FDI and SFO groups. The noninferiority threshold was set to − 0.5 g/dL based on the noninferiority margin and the standard deviation (SD) for change in Hb concentrations (defined as 1.5 g/dL) used in other clinical studies of FDI [18, 19].

We determined that a total of 321 patients, randomized in a 2:1 ratio by minimization, would be required to achieve 80% power at a two-sided significance level of 5%. We estimated that approximately 10% of patients would be excluded from the analyses. Therefore, the final sample size was determined as 360.

### Measurements of clinical parameters

Laboratory tests were performed by LSI Medience Corporation (Tokyo, Japan). Serum hepcidin was measured using the Human Hepcidin Immunoassay Quantikine ELISA kit (R&D Systems Inc., Minneapolis, MN). According to the manufacturer’s instructions, the serum hepcidin level in 40 healthy individuals was 18.4 ± 14.7 ng/mL (mean ± SD).

### Statistical analysis

The mean difference in the maximum change in Hb (g/dL) from baseline between the FDI and SFO groups and the two-sided 95% confidence intervals (CIs) were calculated using an analysis of covariance (ANCOVA) model with treatment group as the independent variable and baseline Hb as a covariate. If the lower bound of the CI was more than -0.5 g/dL, FDI could be declared statistically noninferior to SFO.

The full analysis set (FAS), defined as all patients who received ≥ 1 dose of the study drug and had efficacy data available, was used for all efficacy analyses. Safety analyses were performed using the safety analysis set, defined as all patients who received ≥ 1 dose of the study drug. Baseline demographics were summarized using descriptive statistics. Continuous variables are expressed as mean ± SD and categorical variables are expressed as frequency and proportion. Dosing data are presented as descriptive statistics.

For Hb, MCV, reticulocyte ratio, TSAT, serum ferritin, and hepcidin, descriptive statistics were calculated for the value measured at each analysis visit. For patients whose Hb had increased by ≥ 2 g/dL from baseline and patients with Hb ≥ 12.0 g/dL, the number and percentage of patients reaching these thresholds at each analysis visit and over the treatment period were calculated. The number and percentage of patients who had achieved the cumulative total iron dose during the treatment period were also calculated. Adverse events were coded using the Medical Dictionary for Regulatory Activities (MedDRA), Version 22.0, and any reported treatment-emergent adverse events (TEAEs) were summarized. For serum phosphorus, descriptive statistics are summarized for the values measured at each analysis visit. As a post hoc analysis, the incidence of severe hypophosphatemia (defined as serum phosphorous ≤ 1.0 mg/dL), in line with the threshold set in an overseas study [15], was also calculated.

All statistical analyses were performed using SAS® software version 9.4 (SAS Institute, Cary, NC, USA). P values < 0.05 were considered statistically significant.

## Results

A total of 513 patients were enrolled in this study. Of these, 156 patients (3 patients at the primary registration and 153 patients at the screening visit) were screening failures. A total of 357 patients were finally registered and randomized 2:1 to either the FDI group (238 patients) or the SFO group (119 patients). All patients received at least 1 study treatment except for 1 patient in the FDI group who withdrew consent. Therefore, the safety analysis set included 356 patients (FDI: *n* = 237; SFO: *n* = 119).

**Fig. 2:**
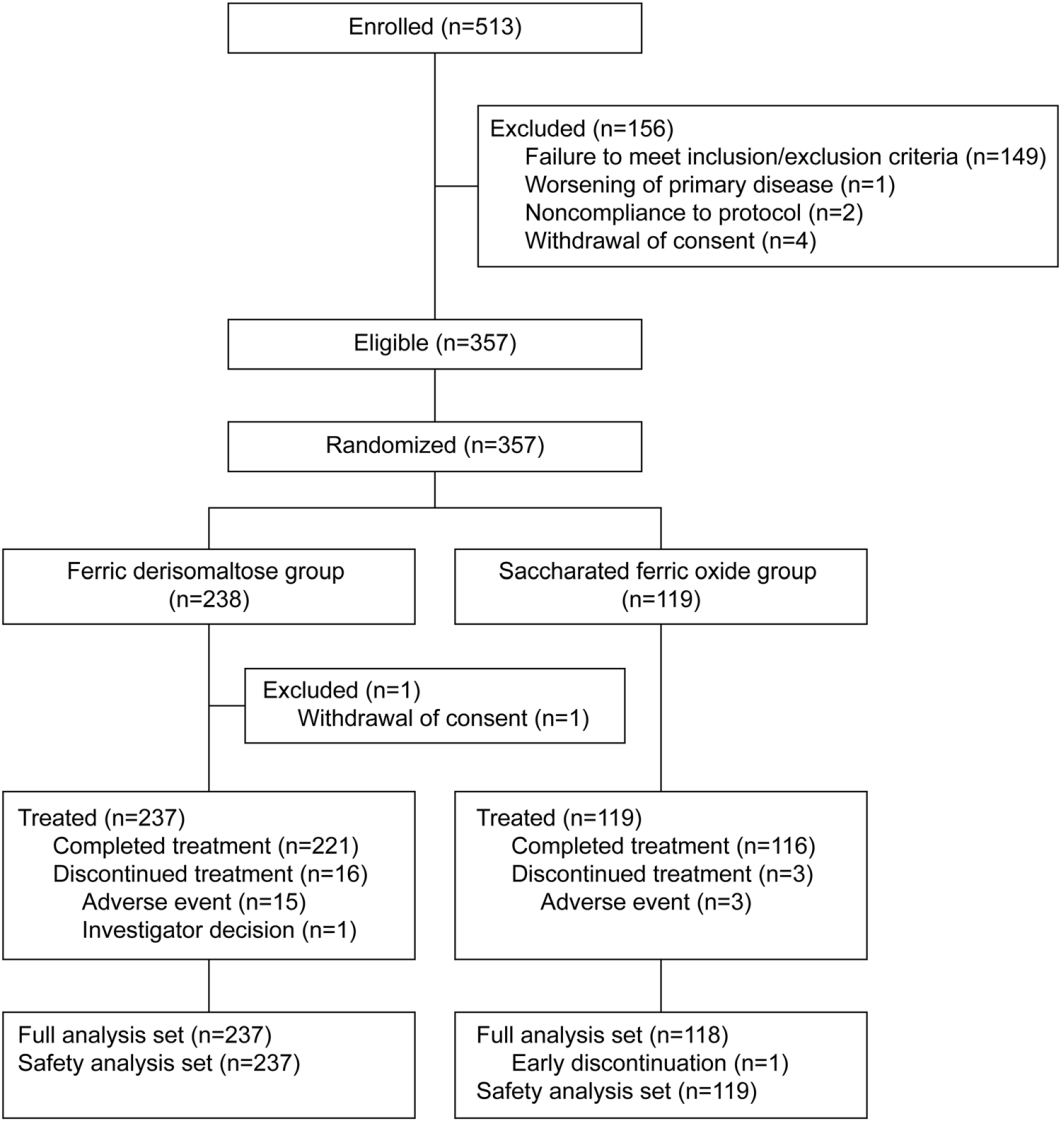
Patient disposition

A total of 19 patients discontinued treatment, representing 6.75% (*n* = 16) of the FDI group and 2.52% (*n* = 3) of the SFO group. The main reasons for discontinuation were TEAEs except for a single patient in the FDI group who was discontinued owing to an investigator decision. Notably, an additional patient in the SFO group was excluded because of the loss in follow-up before the first analysis visit. Therefore, the FAS included 355 patients (FDI: *n* = 237; SFO: *n* = 118). The study disposition of all enrolled patients is presented in Fig. 2.

### Baseline characteristics

The median age of patients was 43 years. Approximately 60% of patients had menorrhagia due to primary disease; the remainder had idiopathic menorrhagia. The most common reason for IV iron injection was the necessity for immediate iron supplementation (83.7%). The mean ± SD Hb concentrations at baseline were 9.12 ± 1.15 g/dL and 8.87 ± 1.29 g/dL in the FDI and SFO groups, respectively. Table 2 presents the baseline characteristics of the 2 treatment groups.

**Table 2:** Demographics and baseline characteristics (full analysis set)

		FDI (*N* = 237)	SFO (*N* = 118)
Age (years)	Mean (SD)	41.5 (6.3)	42.4 (4.9)
	Median	43.0	44.0
	Min–Max	20–49	26–49
*Sex*			
Female	*n* (%)	237 (100.0)	118 (100.0)
Weight (kg)	Mean (SD)	56.29 (8.96)	57.46 (11.02)
	Median	55.40	54.95
	Min–Max	38.5–87.7	41.9–113.5
	< 40	3 (1.3)	0 (0.0)
	40 to < 50	52 (21.9)	25 (21.2)
	50 to < 70	164 (69.2)	82 (69.5)
	≥ 70	18 (7.6)	11 (9.3)
Height (cm)	Mean (SD)	159.7 (5.9)	160.0 (5.6)
	Median	160.0	160.5
	Min–Max	141–175	145–175
BMI (kg/m^2^)	Mean (SD)	22.06 (3.23)	22.45 (4.03)
	Median	21.61	21.27
	Min–Max	15.4–35.2	16.7–43.8
*Cause of menorrhagia*			
Menorrhagia due to primary disease	*n* (%)	146 (61.6)	78 (66.1)
Idiopathic menorrhagia	*n* (%)	91 (38.4)	40 (33.9)
*Reason for intravenous iron injection*			
Intolerant to oral iron preparations within the previous 2 years	*n* (%)	32 (13.5)	16 (13.6)
Unresponsive to oral iron preparations for at least 1 month within the previous 2 years	*n* (%)	6 (2.5)	4 (3.4)
Prompt iron supplementation considered necessary	*n* (%)	199 (84.0)	98 (83.1)
Hemoglobin concentrations (g/dL) at baseline	Mean (SD)	9.12 (1.15)	8.87 (1.29)
	Median	9.20	9.00
	Min–Max	5.0–11.5	5.4–11.4
	< 8	35 (14.8)	31 (26.3)
	8 to < 10	141 (59.5)	57 (48.3)
	≥ 10	61 (25.7)	30 (25.4)
eGFR (mL/min/1.73 m^2^) at baseline	Mean (SD)	85.26 (13.53)	84.63 (13.27)
	Median	84.10	83.50
	Min–Max	61.3–139.5	59.4–130.2
	< 60	0 (0.0)	1 (0.8)
	60 to < 90	161 (67.9)	82 (69.5)
	≥ 90	76 (32.1)	35 (29.7)

Percentages are based on *n*, the total number of patients in the treatment group for whom the information is available. The baseline value is the value measured before administration of the study treatment

*BMI* body mass index, *eGFR* estimated glomerular filtration rate, *FDI* ferric derisomaltose, *max* maximum, *min* minimum, *SD* standard deviation, *SFO* saccharated ferric oxide

### Exposure

The total doses (mean ± SD) administered were 1226.4 ± 341.9 mg and 1103.1 ± 291.0 mg in the FDI and SFO groups, respectively. The mean ± SD number of administrations was 1.65 ± 0.49 and 9.72 ± 2.54 in the FDI and SFO groups, respectively. The percentage of patients who achieved the cumulative iron dose during the 8-week treatment period was 92.8% in the FDI group and 43.2% in the SFO group.

### Endpoints

#### Efficacy

**Fig. 3:**
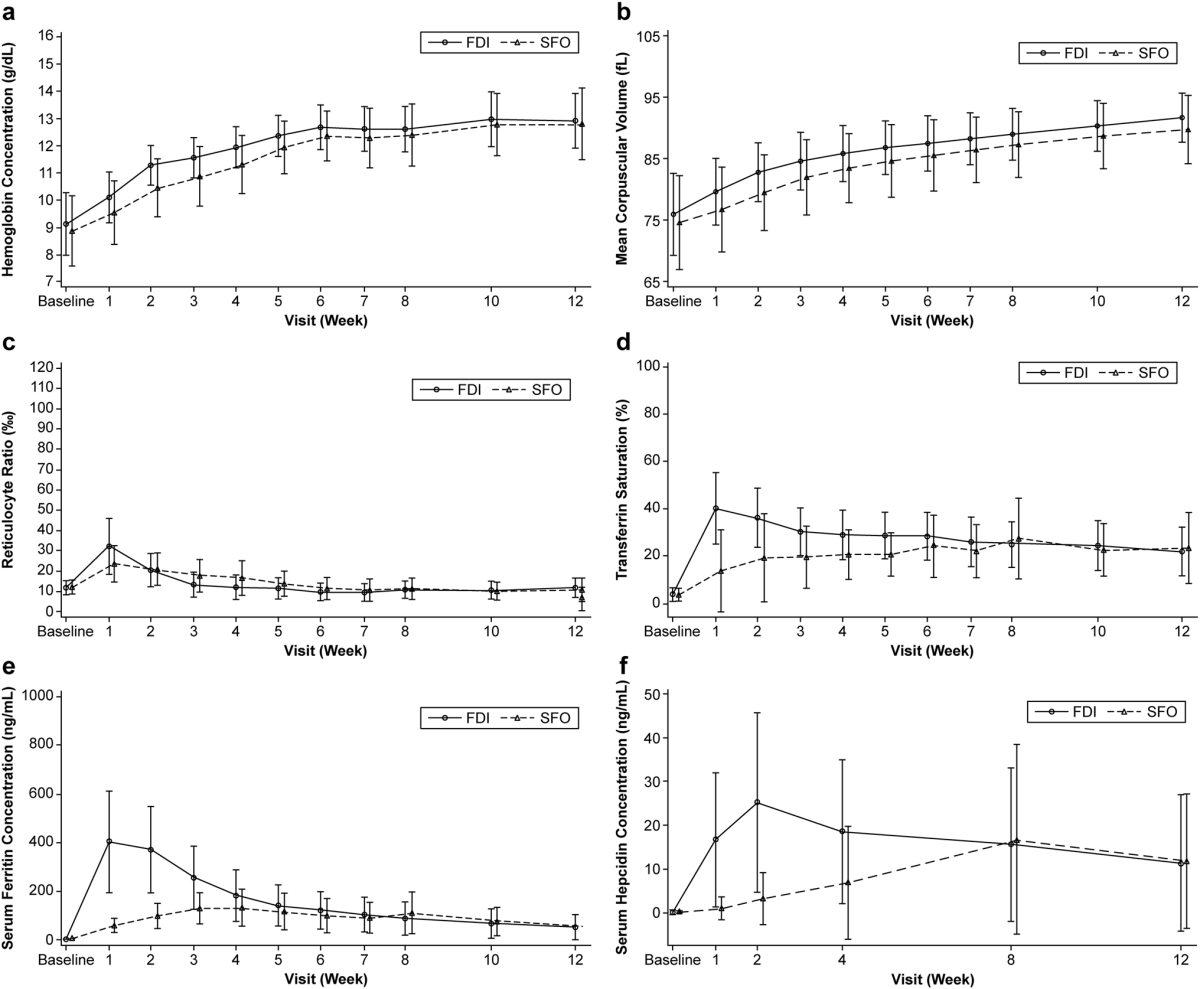
Laboratory value changes over the study period (mean ± SD) in (a) hemoglobin concentration (g/dL), (b) mean corpuscular volume (fL), (c) reticulocyte ratio (‰), (d) transferrin saturation (%), (e) serum ferritin concentration (ng/mL), and (f) serum hepcidin concentration (ng/mL). *FDI* ferric derisomaltose, *SD* standard deviation, *SFO* saccharated ferric oxide.

After treatment initiation at Week 0, mean Hb concentrations increased steadily in both groups. The mean ± SD maximum post-baseline Hb concentrations were 13.38 ± 0.83 g/dL and 13.27 ± 0.99 g/dL in the FDI and SFO groups, respectively. The difference in least-squares means in the maximum changes in Hb concentrations from baseline between the FDI and SFO groups was 0.06 (95% CI − 0.13, 0.24) g/dL (Table 3). The mean change in Hb concentration from baseline was higher in the FDI group than in the SFO group until Week 5. The mean Hb concentration reached over 12 g/dL in the FDI group at week 5, 1 week earlier than in the SFO group. The mean ± SD Hb concentration at week 6 was 12.66 ± 0.83 g/dL in the FDI group and 12.35 ± 0.92 g/dL in the SFO group and remained over 12 g/dL in both groups thereafter (Fig. 3a). The percentage of patients whose Hb concentration had increased by ≥ 2 g/dL from baseline increased over time in both treatment groups, except for a temporary decrease observed at week 7 in the SFO group. The percentage of such patients exceeded 50% at an earlier time point in the FDI group (at week 2) than in the SFO group (at week 3). The percentage of patients with Hb ≥ 12.0 g/dL also increased over time in both treatment groups, except for a temporary decrease at week 7 in the SFO group, and it increased earlier in the FDI group than in the SFO group. Figure 3a presents Hb values at each study visit.

**Table 3:** Maximum change from baseline in hemoglobin concentrations (g/dL) (ANCOVA)

Treatment group	LS means			Difference in LS means (FDI—SFO)		
	Estimate	SE	95% confidence interval	Estimate	SE	95% Confidence interval
FDI (*n* = 237)	4.33	0.05	4.22, 4.44	0.06	0.10	− 0.13, 0.24
SFO (*n* = 118)	4.27	0.08	4.12, 4.42			

The baseline value is the value measured before administration of the study treatment

*n* = the total number of patients in the treatment group for whom the information is available

Maximum change was defined as the maximum value for change from baseline when measured from study day 2 to study day 91

Maximum change from baseline was analyzed using an ANCOVA model with treatment group as the independent variable and baseline hemoglobin as a covariate

If the lower bound of the confidence interval in difference in LS means was more than − 0.5 g/dL, then FDI was declared to be statistically noninferior to SFO

*ANCOVA* analysis of covariance, *FDI* ferric derisomaltose, *LS* least-squares, *SE* standard error, *SFO* saccharated ferric oxide

The mean ± SD MCV values at baseline were 75.9 ± 6.7 fL in the FDI group and 74.6 ± 7.6 fL in the SFO group. The mean MCV levels increased over time in both treatment groups, and the maximum values were observed at week 12 (91.7 ± 4.0 fL vs 89.7 ± 5.6 fL, respectively) (Fig. 3b).

The mean ± SD reticulocyte ratios at baseline were 11.6 ± 3.2‰ in the FDI group and 12.1 ± 3.5‰ in the SFO group. In both groups, the ratio peaked at week 1. However, the reticulocyte ratio at week 1 was greater in the FDI group (32.0 ± 13.8‰) than in the SFO group (23.5 ± 8.8‰) (Fig. 3c).

The mean ± SD TSAT values at baseline were 3.9 ± 2.8% in the FDI group and 3.9 ± 2.6% in the SFO group. The maximum mean change in TSAT from baseline was observed at week 1 in the FDI group and week 8 in the SFO group. The mean ± SD TSAT values were comparable between the FDI and SFO groups at Week 12 (21.9 ± 10.2% vs 23.5 ± 15.0%, respectively) and maintained at approximately 20% throughout the follow-up period in both treatment groups (Fig. 3d).

The mean ± SD serum ferritin levels at baseline were 3.56 ± 2.40 ng/mL in the FDI group and 3.11 ± 1.64 ng/mL in the SFO group. The mean serum ferritin level peaked at week 1 and then gradually decreased in the FDI group. In contrast, in the SFO group, serum ferritin levels gradually increased until week 4 and then remained about the same for the remainder of the study period (Fig. 3e).

#### Exploratory

Serum hepcidin concentration (mean ± SD) in the FDI group peaked at week 2 (25.2 ± 20.0 ng/mL) and then gradually decreased until week 12, while in the SFO group, it increased slowly until Week 8 (16.8 ± 21.7 ng/mL) and decreased at week 12. The week 12 hepcidin value was similar in both treatment groups (FDI group: 11.4 ± 15.6 ng/mL vs SFO group: 11.8 ± 15.4 ng/mL) (Fig. 3f).

#### Safety

Both TEAEs (66.2% vs 90.8%) and treatment-related TEAEs (46.8% vs 87.4%) were lower in the FDI group than in the SFO group. Table 4 shows TEAEs occurring in ≥ 5% of patients in either treatment group, which included nasopharyngitis (14.3% vs 15.1% in the FDI and the SFO groups, respectively), pyrexia (11.4% vs 2.5%, respectively), urticaria (8.9% vs 0.8%, respectively), hypophosphatemia (5.9% vs 55.5%, respectively), headache (5.9% vs 5.0%, respectively), serum ferritin increase (5.5% vs 0.8%, respectively), and blood phosphorus decrease (1.3% vs 25.2%, respectively) (Table 4). The percentage of patients who met treatment interruption criteria was much lower in the FDI group (32.5%) than in the SFO group (83.2%). The percentage of patients who had a serum phosphorus level < 2.0 mg/dL was lower in the FDI group (8.4%) than in the SFO group (83.2%), while the percentage of patients who had a serum ferritin value ≥ 500 ng/mL was higher in the FDI group (27.0%) than in the SFO group (0.8%). No TEAEs leading to death were reported. Two patients (0.8%) in the FDI group experienced 1 serious TEAE each (contusion and colon cancer). No serious treatment-related TEAEs were reported. TEAEs leading to treatment interruption were reported among 1.7% of patients in the FDI group and 72.3% of patients in the SFO group. More patients reported TEAEs leading to drug withdrawal in the FDI group (6.3%) than in the SFO group (2.5%). Urticaria (3.0%) and pyrexia and rash (1.3% each) were the most common TEAEs leading to drug withdrawal in the FDI group.

**Table 4:** Adverse events (safety analysis set)

Preferred term	TEAE		Treatment-related TEAE	
	FDI (*N* = 237)	SFO (*N* = 119)	FDI (*N* = 237)	SFO (*N* = 119)
Number of patients with at least one TEAE	157 (66.2)	108 (90.8)	111 (46.8)	104 (87.4)
Nasopharyngitis	34 (14.3)	18 (15.1)	0 (0.0)	0 (0.0)
Pyrexia	27 (11.4)	3 (2.5)	20 (8.4)	2 (1.7)
Urticaria	21 (8.9)	1 (0.8)	19 (8.0)	0 (0.0)
Hypophosphatemia	14 (5.9)	66 (55.5)	14 (5.9)	66 (55.5)
Headache	14 (5.9)	6 (5.0)	10 (4.2)	4 (3.4)
Serum ferritin increased	13 (5.5)	1 (0.8)	13 (5.5)	1 (0.8)
Blood phosphorus decreased	3 (1.3)	30 (25.2)	3 (1.3)	30 (25.2)

Medical Dictionary for Regulatory Activities Version 22.0

Data are *n* (%)

*FDI* ferric derisomaltose, *SFO* saccharated ferric oxide, *TEAE* treatment-emergent adverse event

**Fig. 4:**
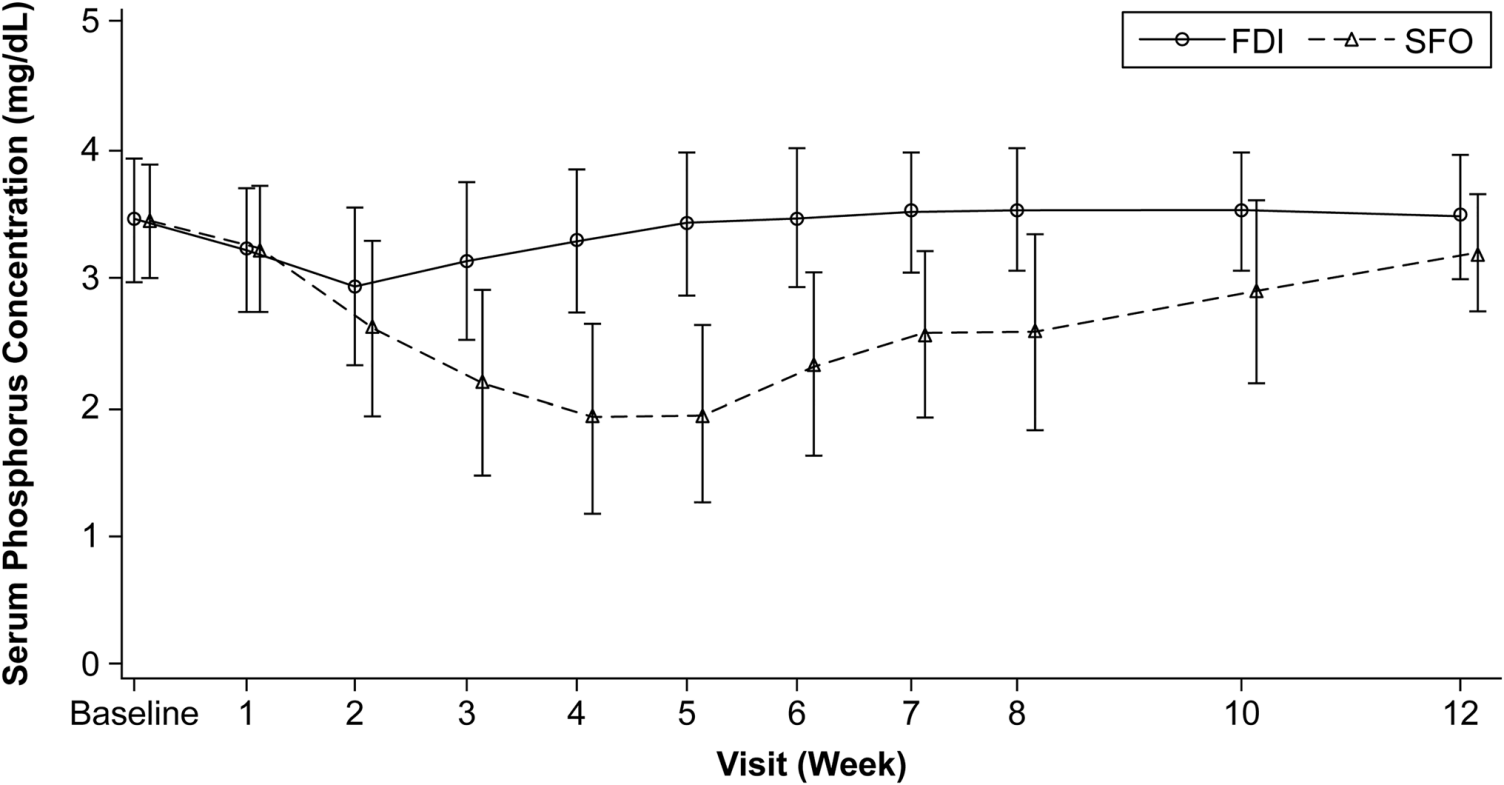
Laboratory value changes over the study period (mean ± SD) in serum phosphorus (mg/dL). *FDI* ferric derisomaltose, *SD* standard deviation, *SFO* saccharated ferric oxide.

Serum phosphorus values at baseline (mean ± SD) were 3.45 ± 0.48 mg/dL in the FDI group and 3.44 ± 0.44 mg/dL in the SFO group. In the FDI group, the mean serum phosphorus values were maintained at ≥ 2.5 mg/dL until Week 12, while in the SFO group, they gradually decreased until week 4 and were < 2.5 mg/dL from week 3 to week 6. The mean ± SD serum phosphorus values at week 12 were 3.48 ± 0.48 mg/dL and 3.19 ± 0.45 mg/dL in the FDI and SFO groups, respectively (Fig. 4). Severe hypophosphatemia (≤ 1.0 mg/dL) occurred in 6.7% of SFO-treated patients compared with none of the FDI-treated patients.

## Discussion

The primary endpoint (maximum change in Hb concentration from baseline) was noninferior for the FDI group compared with the SFO group. The percentage of patients whose Hb concentration had increased by ≥ 2 g/dL and those with Hb concentration of ≥ 12.0 g/dL, as the secondary endpoints, increased earlier in the FDI group than in the SFO group. The reticulocyte ratio was also higher at Week 1 in the FDI group than in the SFO group, suggesting that the iron was rapidly utilized by erythropoiesis, resulting in the rapid increase in Hb concentration. This finding could be attributed to the early completion of the administration of the cumulative iron doses in the FDI group. Of note, the percentage of patients who achieved the cumulative total iron dose during the 8-week treatment period was higher in the FDI group than in the SFO group, although the mean total doses administered were comparable between the groups. Many of the patients could not receive the total iron dose because of TEAEs leading to treatment interruption (i.e., prolonged hypophosphatemia) and not because of insufficient duration of the treatment period.

An increase in serum ferritin was observed immediately after FDI administration. Furthermore, at different instances, 64 patients (27.0%) manifested serum ferritin levels ≥ 500 ng/mL, the threshold for iron overload according to the Japanese guidelines [5]. Although the serum ferritin value can be a useful index for the amount of iron stored in the body, its level immediately after the administration of IV iron can be misleading. Because serum ferritin is derived primarily from macrophages [20], its level essentially reflects the amount of stored iron in the reticuloendothelial macrophages. In fact, by week 3 (i.e., 2 weeks after the last administration), the mean serum ferritin value in the FDI group had returned to the normal range (25–250 ng/mL) defined in the guidelines, and no TEAEs associated with iron overload were reported. Thus, the remarkable increase in serum ferritin observed in this study was probably a transient event with minimal clinical significance, reflecting rapid incorporation of IV iron into macrophages. Iron supplemented from IV iron preparations is temporarily stored in reticuloendothelial cells, such as macrophages [21], and is gradually (over approximately 2 weeks) used for hematopoiesis [22]. By week 12 (i.e., the day of the last observation), the systemic iron appeared to have been appropriately restored as measured by the mean serum ferritin value.

As an exploratory endpoint, we investigated serum hepcidin levels throughout the study period. Hepcidin is the principal regulatory hormone that mediates the homeostasis of extracellular iron concentrations [23]. Expression of hepcidin is upregulated by iron loading and inflammatory cytokines (especially interleukin 6) and downregulated by iron deficiency, hypoxia, anemia, and erythroferrone produced by erythroid cells [24]. Serum hepcidin concentration reached its maximum in both groups after the cumulative total iron dose was achieved. The maximum serum hepcidin concentration was accompanied by increases in levels of both serum ferritin and TSAT, which reflected systemic iron status, and then gradually decreased over time.

The safety results from this study demonstrated an acceptable safety profile for FDI in the treatment of IDA associated with menorrhagia. The incidence of TEAEs was lower in the FDI group than in the SFO group. However, the incidences of pyrexia and urticaria were higher among patients in the FDI group than in the SFO group. Most of these TEAEs were reported at week 1. Although most were mild and all resolved by the end of the study period, their occurrence reinforces the need for careful observation even when administering IV FDI.

The percentage of patients who had serum phosphorus level < 2.0 mg/dL was remarkably lower in the FDI group (8.4%) than in the SFO group (83.2%). Severe hypophosphatemia (≤ 1.0 mg/dL) also occurred in 6.7% of SFO-treated patients compared with none of the FDI-treated patients. Acute hypophosphatemia can affect muscles and neurons, and can lead to respiratory failure, fatigue, tremors, malaise, generalized weakness, neuropathy, irritability, and convulsions. Chronic hypophosphatemia affects the skeleton, leading to osteomalacia and fracture, muscle weakness, and eventual sarcopenia. In children, rickets and growth retardation can occur [25]. Various potential mechanisms have been proposed to explain IV iron-mediated hypophosphatemia, including the direct nephrotoxic effect of IV iron leading to increased renal loss of phosphate [26, 27], and increased cellular uptake of extracellular phosphate in association with the rapid expansion of erythropoiesis [28]. More recently, another mechanism underlying hypophosphatemia has been demonstrated. Active, intact FGF23, a key component in phosphate regulation, increases after treatment with certain IV iron preparations, leading to increased urinary excretion of phosphate and, thus, hypophosphatemia [6, 29, 30]. Besides its effects as a phosphaturic hormone, FGF23 also inhibits the activation of 25-(OH) vitamin D_3_ to 1,25-(OH)_2_ vitamin D_3_ (calcitriol), thereby potentially explaining the mild hypocalcemia and subsequent increase in circulating parathyroid hormone (PTH) following IV iron treatment. Due to the phosphaturic effects of PTH, this mechanism may prolong hypophosphatemia after FGF23 returns to normal levels [31]. Notably, in randomized controlled studies [12, 32] and a meta-analysis [31], FDI was associated with significantly lower rates of hypophosphatemia than ferric carboxymaltose (FCM), another IV iron preparation that was recently approved in Japan. High rates of hypophosphatemia after treatment with SFO have also been observed in a randomized controlled study comparing SFO with FCM [33]. Currently, the mechanism of preparation-specific risks for hypophosphatemia is not fully understood, but the carbohydrate backbones of certain iron formulations, including FCM, may inhibit the cleavage of intact FGF23, leading to renal phosphate loss and hypophosphatemia [25]. According to recent recommendations by the European Medicine Agency’s pharmacovigilance risk assessment committee and the US Food and Drug Administration, the risks associated with FCM therapy should be carefully considered in patients with known bone disease or high fracture risk [34].

This study has 3 main limitations. First, the study sample was limited to Japanese premenopausal women with IDA associated with menorrhagia. Therefore, the efficacy and safety of FDI for the treatment of IDA in postmenopausal women and Japanese men of all ages is unknown. Second, in the current study, the first administration of FDI was performed only through IV drip infusion, and bolus injection was allowed only for the second administration. However, in other countries, either an IV drip infusion or a bolus injection can be used for the first administration. Therefore, the efficacy and safety of an IV bolus injection for the first administration in Japanese patients needs to be evaluated. Finally, we excluded patients with renal impairment potentially associated with renal anemia (estimated glomerular filtration rate < 60 mL/min/1.73 m^2^). Therefore, studies are needed to evaluate the efficacy and safety of FDI among patients with renal impairment.

In conclusion, the primary endpoint, the noninferiority of FDI compared with SFO in terms of the maximum change in Hb concentration from baseline, was demonstrated. A higher dose of FDI per infusion was tolerated, with a lower incidence of hypophosphatemia, compared with SFO. This would result in fewer medical visits and a more rapid increase in Hb with fewer TEAEs. These results were essentially consistent with those of previous studies conducted in other countries [35–38]. Thus, the benefits of FDI with respect to the number of patient visits and healthcare system resources spent on infusion recommend its consideration as a new option for the treatment of IDA in Japanese patients.

## Supplementary Information

Below is the link to the electronic supplementary material.

Supplementary file1 (DOCX 19 KB)
