# Correction to: Intravenous ferric derisomaltose versus saccharated ferric oxide for iron deficiency anemia associated with menorrhagia: a randomized, open-label, active-controlled, noninferiority study

**DOI:** 10.1007/s12185-022-03485-8

**Published:** 2022-11-08

**Authors:** Hiroshi Kawabata, Takeshi Tamura, Soichiro Tamai, Akiko Fujibayashi, Motoi Sugimura, Study Group, Jun Hayakawa, Hisato Oku, Yoshiaki Ota, Sonoe Nishiguchi, Kiyohiko Yamada, Masayasu Nomura, Toshiro Mizutani, Yoshihiro Tamura, Kyoka Amemiya, Mamoru Urabe, Hirofumi Henmi, Kozo Aisaka, Atsuya Fujito, Masataka Oku, Chisei Tei, Akinori Kawata, Masaya Hirose, Masuo Yoshioka, Chizue Nishizawa, Ikuyo Horiguchi, Kozo Hirai, Akiko Tanabe, Shohei Yoshida, Yoshihiro Umezawa, Yuji Kashiwazaki, Hideki Kamegai, Toshio Saito, Kazutoshi Naritaka, Shigehito Yamauchi, Kenji Akazawa, Koji Kobiki, Hiroshi Tsujioka, Yukari Sumi, Reiko Matsumoto, Mari Kiuchi, Yukari Utsugisawa, Masanori Maruyama, Hiroyuki Furumoto, Kazuhiro Minegishi, Masao Takane, Asuka Yoshii, Tsuneo Yokokura, Hideki Hanashi, Sumie Yukawa

**Affiliations:** 1 Department of Hematology, National Hospital Organization Kyoto Medical Center, Kyoto, Japan; 2 Clinical Development Department, Nippon Shinyaku Co., Ltd, Kyoto, Japan; 3 Data Science Department, Nippon Shinyaku Co., Ltd, Kyoto, Japan; 4 Department of Obstetrics, Gynecology and Family Medicine, Hamamatsu University School of Medicine, Hamamatsu, Shizuoka Japan

## Correction to: International Journal of Hematology (2022) 116:647–658 10.1007/s12185-022-03401-0

In the original publication of the article, the Fig. 3 was published with errors. The correct Fig. 3 is given in this correction.

**Fig. 3:**
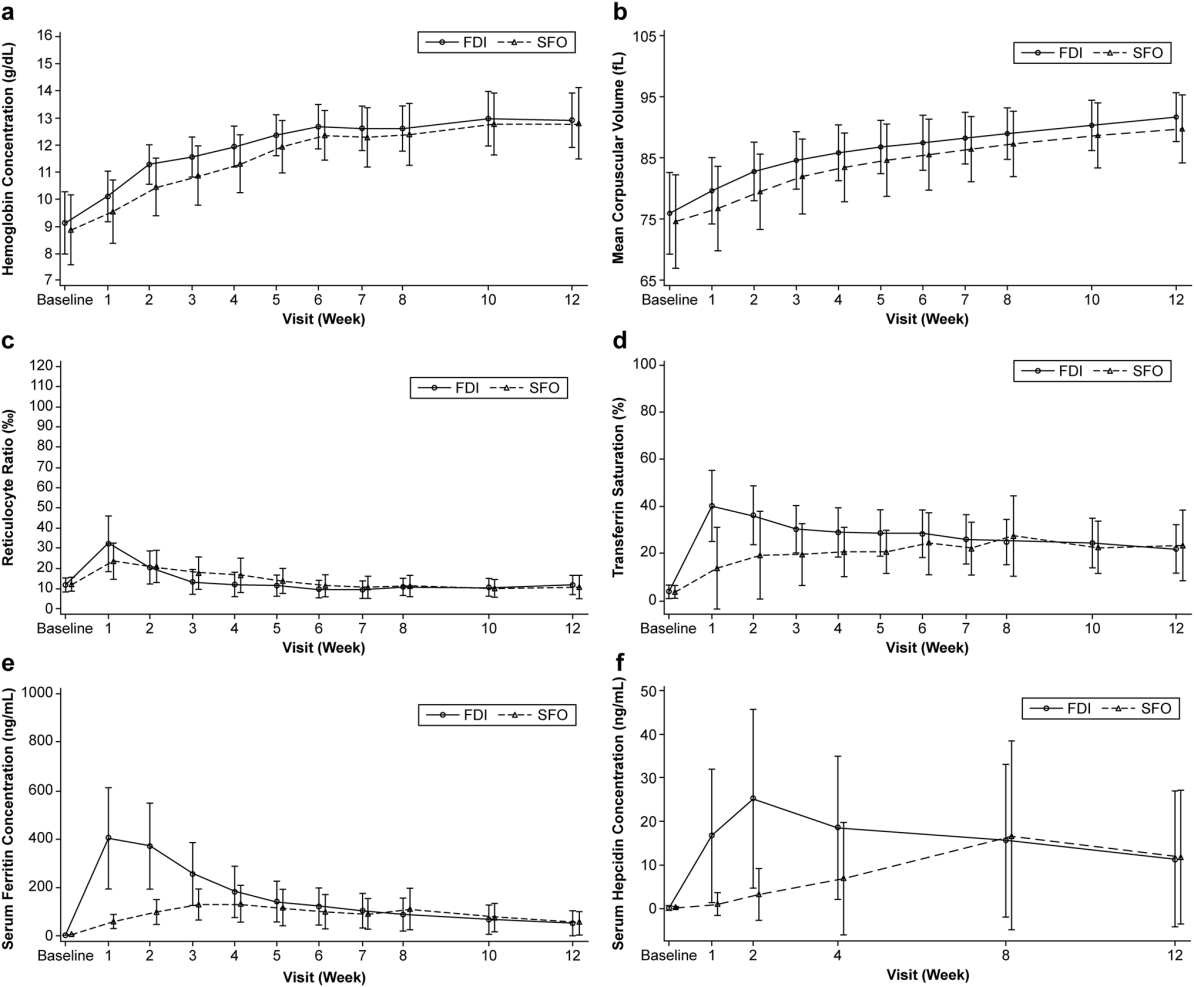
Laboratory value changes over the study period (mean ± SD) in (a) hemoglobin concentration (g/dL), (b) mean corpuscular volume (fL), (c) reticulocyte ratio (‰), (d) transferrin saturation (%), (e) serum ferritin concentration (ng/mL), and (f) serum hepcidin concentration (ng/mL). *FDI* ferric derisomaltose, *SD* standard deviation, *SFO* saccharated ferric oxide.

