# Supplementary material for: Intravenous ferric derisomaltose versus saccharated ferric oxide for iron deficiency anemia associated with menorrhagia: a randomized, open-label, active-controlled, noninferiority study
Source: Int J Hematol. 2022 Jul 6;116(5):647–58. doi: 10.1007/s12185-022-03401-0 (PMC9588477; doi:10.1007/s12185-022-03401-0)
Supplement: Supplementary file 1 — Supplementary file1 (DOCX 19 KB) [file 12185_2022_3401_MOESM1_ESM.docx]

**Electronic Supplementary Material**

***International Journal of Hematology***

**Intravenous ferric derisomaltose versus saccharated ferric oxide for iron deficiency anemia associated with menorrhagia: A randomized, open-label, active-controlled, noninferiority study**

**Author Names and Affiliations**

Hiroshi Kawabata^1^, Takeshi Tamura^2^, Soichiro Tamai^3^, Akiko Fujibayashi^2^, Motoi Sugimura^4^, and Study Group^5^

^1^Department of Hematology, National Hospital Organization Kyoto Medical Center, Kyoto, Japan

^2^Clinical Development Department, Nippon Shinyaku Co., Ltd., Kyoto, Japan

^3^Data Science Department, Nippon Shinyaku Co., Ltd., Kyoto, Japan

^4^Department of Obstetrics, Gynecology and Family Medicine, Hamamatsu University School of Medicine, Hamamatsu, Shizuoka, Japan

^5^See Online Resource 1

**Corresponding Author**:

Hiroshi Kawabata, MD, PhD

Email: hkawabat@kuhp.kyoto-u.ac.jp

**Study group**

Jun Hayakawa, Hayakawa Clinic (Osaka)

Hisato Oku, Chayamachi Ladies Clinic

Yoshiaki Ota (Mar 2019-Dec 2019), Mari Sawada (Dec 2019-present), Kurashiki Medical Clinic

Sonoe Nishiguchi, Asahi Clinic (Kagawa)

Kiyohiko Yamada, Kokikai Tsujinaka Hospital Kashiwanoha

Masayasu Nomura, Nomura Clinic Namba

Toshiro Mizutani, Aiiku Ladies Clinic

Yoshihiro Tamura, Shizuoka Saiseikai General Hospital

Kyoka Amemiya, Itami City Hospital

Mamoru Urabe, Kusatsu General Hospital

Hirofumi Henmi, Tonan Hospital

Kozo Aisaka, Hamada Hospital

Atsuya Fujito, Fujito Clinic

Chisei Tei, Sei Women's Clinic

Akinori Kawata, JOHAS Kagawa Rosai Hospital

Masaya Hirose, Hyogo Prefectural Amagasaki General Medical Center

Masuo Yoshioka, Suzuran Clinic

Chizue Nishizawa, Nagano Municipal Hospital

Kozo Hirai, Minami Morimachi Ladies' Clinic

Akiko Tanabe, Tanabe Ladies' Clinic

Shohei Yoshida, Ginza Yoshida Iin

Yoshihiro Umezawa, Den-en-tyofu Family Clinic

Yuji Kashiwazaki, Kashiwazaki Obstetrics and Gynecology Clinic

Hideki Kamegai, Osakafu Saiseikai Suita Hospital

Toshio Saito, Funabashi Municipal Medical Center

Shigehito Yamauchi, Aijinkai Ota General Hospital

Kenji Akazawa, Akazawa Clinic

Koji Kobiki, Kobiki Women's Clinic

Hiroshi Tsujioka, Aso Co., Ltd., Iizuka Hospital

Yukari Sumi, Toranomon Women’s Clinic

Reiko Matsumoto, Chiba Aoba Municipal Hospital

Mari Kiuchi, Ryoshukai Kanauchi Medical Clinic

Yukari Utsugisawa, Yokohama Motomachi Women's Clinic LUNA

Masanori Maruyama, Jiseikai Maruyama Memorial General Hospital

Hiroyuki Furumoto, Tokushima Municipal Hospital

Kazuhiro Minegishi, Hiroo Minegishi Obstetrics & Gynecology Clinic

Masao Takane, Takane Medical Clinic

Asuka Yoshii (Mar 2019-Aug 2019), Yoshiaki Uchiyama (Aug 2019-present), Parkside Hiroo Ladies Clinic

Tsuneo Yokokura, Yokokura Clinic

Hideki Hanashi, Seikokai New Medical Research System Clinic

Sumie Yukawa, Yukawa Women's Clinic

These affiliations are at the time of the conduct of the study.
